# PReS-FINAL-2268: Sarcoidosis in children seen at the pediatric rheumatology clinics of two referral hospitals in Cape Town, South Africa

**DOI:** 10.1186/1546-0096-11-S2-P258

**Published:** 2013-12-05

**Authors:** LO Okong'o, C Scott, K Webb

**Affiliations:** 1 Paediatric Rheumatology, Red Cross War Memorial Children's Hospital, Cape Town, South Africa; 2 School of Child and Adolescent Health, University of Cape Town, Cape Town, South Africa

## Introduction

Sarcoidosis is a relatively uncommon condition in children. Reports from multiracial societies such as the USA indicate that sarcoidosis is more common in Africans than other racial groups. However, few reports of cases of childhood Sarcoidosis have been published from sub-Saharan Africa to shed light on the burden of sarcoidosis and the demographics and clinical presentation of children diagnosed with sarcoidosis.

## Objectives

To describe the occurrence and clinical presentation of Sarcoidosis among children seen at the rheumatology clinics in two referral hospitals in Cape Town, South Africa.

## Methods

We conducted a search for patients with a diagnosis of sarcoidosis in the pediatric rheumatology data base of the two referral hospitals affiliated to the University of Cape Town. The proportion of the patients with a diagnosis of sarcoidosis, their demographic and clinical features was then determined.

## Results

A total of 251 Patients were in the data base by the time of the review; 4 (1.6%) had a diagnosis of sarcoidosis. They were aged 10, 4, 17 and 15 years at study time point; 4, 3.5, 11 and 14.5 years at onset of symptoms; and 10, 3.5, 16 and 14.5 years respectively at diagnosis. Three were female. Of the 4 patients, 2 were black and 2 colored. The 1^st ^child presented with recurrent acute uveitis from the age of 4 years and iris nodules (probable ocular sarcoidosis). The second had acute respiratory distress, lymphadenopathy and hemophagocytic lymphohistiocytosis. Both had raised serum Angiotensin Converting Enzyme (ACE) levels. The 3^rd ^patient had polyarthritis and skin nodules. The 4^th^, an HIV positive boy, presented with respiratory distress, skin lesions and neuropathy (diaphragmatic paralysis and foot drop). The second, 3^rd ^and 4^th ^patients had non caseating granulomas on tissue biopsy suggestive of sarcoidosis.

Three of the 4 patients (only 1 of whom had positive tuberculin skin test) were treated for TB in the course of their illness before Sarcoidosis was identified as the cause of the symptoms. This was despite attempts to identify mycobacteria by microscopy, culture and GeneXpert on body fluids and biopsy specimen yielding no evidence of mycobacterial infection. The decision to treat for TB was mainly based on clinical and abnormal chest radiograph findings. Treatment for sarcoidosis was instituted with prednisone and methotrexate in 3 patients and methotrexate alone (with topical ophthalmic steroids) in 1 patient. Three of the patients have shown good response to treatment while the patient with polyarthritis and skin nodules has had an unremitting disease course.

## Conclusion

Though Sarcoidosis is a rare disease in children, it still constitutes a significant proportion of pediatric rheumatology consultations. Children with sarcoidosis may present with clinical and radiological features similar to TB presenting a challenge to clinicians on differentiating them especially in the high TB burden countries in sub Saharan Africa. However, where clinical, laboratory and radiological investigations do not fully support TB infection in TB suspects, Sarcoidosis and other granulomatous inflammatory conditions should be considered and be investigated appropriately.

## Disclosure of interest

None declared.

